# Effects of SARS-CoV-2 vaccination on blood donation and blood banks in India

**DOI:** 10.1016/j.amsu.2022.103772

**Published:** 2022-05-11

**Authors:** Reem Hunain, Utkarsha Uday, Sudhan Rackimuthu, Faisal A. Nawaz, Kapil Narain, Mohammad Yasir Essar, Majeeb ur Rehman, Shoaib Ahmad, Ayesha Butt

**Affiliations:** a Kasturba Medical College, Manipal, India; b West Bengal University of Health Sciences, Kolkata, India; c Father Muller Medical College, Mangalore, Karnataka, India; d College of Medicine, Mohammed Bin Rashid University of Medicine and Health Sciences, Dubai, United Arab Emirates; e Nelson R. Mandela School of Medicine, University of KwaZulu-Natal, Durban, South Africa; f Kabul University of Medical Sciences, Kabul, Afghanistan; g Punjab Medical College, Faisalabad, Punjab, Pakistan; h Section of Hematology, Yale University School of Medicine, Connecticut, USA

**Keywords:** Blood donation, COVID-19, India, COVID-19, coronavirus disease 19, DGHS, directorate general of health services, FDA, food and drug administration, mRNA, messanger ribonucleic acid, NBTC, national blood transfusion council, RT-PCR, reverse transcription polymerase chain reaction, SARS-CoV-2, severe acute respiratory syndrome -2, UK, United Kingdom

## Abstract

India, the second most populous country in the world, started its mass vaccination campaign on January 16th, 2021. With the aim to vaccinate 1.3 billion people, this vaccination programme was dubbed as the world's largest vaccination drive. However, with depleted blood stores due to the COVID-19 pandemic and lockdown leading to reduced blood camps, the superposed regulations on blood donation deferral poses an impending risk of depletion of blood and its products. This will lead to the inability in meeting unpredictable patterns of demand in blood requirement post-pandemic. Hence to prevent avoidable risks of blood shortage in surgeries and lifesaving procedures, a secure storage system should be ensured.

Dear Editor,

The second wave of severe acute respiratory syndrome - Coronavirus - 2 (SARS-CoV-2) pandemic had a disastrous aftermath on India, the most populous democracy in the world. Its effects were devastating surpassing the number of new daily cases and deaths compared to the first wave, with daily confirmed cases exceeding two hundred thousand on April 14, 2021 [1]. Global analysis of herd immunity in Coronavirus disease 2019 (COVID-19) has shown the urgent need for COVID-19 vaccination efforts in order to curb the pandemic [2]. On January 16, 2021, India began the world's largest vaccination programme for COVID-19, prioritizing the healthcare and frontline workers, followed by geriatric population age and 45–59 years aged people with comorbidities [3].

The National Blood Transfusion Council (NBTC) of India issued guidelines on March 5, 2021, deferring SARS-CoV-2 vaccinated citizens from donating blood for 28 days post vaccination [4]. This meant deferral for 56 days for Covaxin and 90 days for Covishield, after the first jab, which led to a widespread concern regarding the blood donation camps and availability of blood components. Hence, donation of blood before getting vaccinated was widely recommended. The directorate general of health services (DGHS), on May 5, 2021, reducedthis period to14 days post vaccination and 15 days after a negative reverse transcription polymerase chain reaction (RT-PCR) for COVID-19 [4].

According to the NBTC, there are 2023 blood banks in India which receive 78% blood from voluntary donors. The land of 1.2 billion peopleneeds 12 million units of blood annually but hardly collects 9 million which is a 25% deficit during non-COVID times, as estimated by the ministry of health and family welfare (MoHFW).However, this year's situation was further worsened due to skyrocketing COVID-19 cases, which have led to a substantial reduction by 50% in the number and scale of blood donation camps organised [5]. Vaccination for the 18 to 45 age group, which comprises the majority of blood donors, can cause massive disruption in the blood donation system in India. Furthermore, nationwide lockdown, fear of transmission of SARS-CoV-2 virus via blood, less demand and hence less supply due to reduced number of elective surgeries and employee absenteeism due to isolation add to probable causes. Limited shelf life of 35–49 days for blood and blood components has also led to increased rates of discarded unused blood products due to expiry [6].

This deficit in blood donation stores could potentially be further exacerbated by the mass vaccination starting for 18 to 44-year age groups. Individuals who receive live vaccines are usually deferred temporarily from donating blood due to potential risk for transferring the infection through blood and blood products [7]. Extensive literature searches failto identify any evidence of transmission via transfusion of blood or blood components and no cases have been reported [8]. On the other hand, the pandemic has had a negative impact on blood supplies through reduced blood donation and reduced availability of appropriate collection facilities because of operational disruptions [9].

International regulatory bodies like Food and Drug Administration (FDA) had a deferral period of 14 days post vaccination in case of live-attenuated viral vaccine or if the personis symptomatic. However, in individuals with a non-replicating, inactivated, or messanger ribonucleic acid (mRNA) -based COVID-19 vaccine, blood donation is allowed without a waiting period [10]. In the United Kingdom (UK), vaccines currently licensed are not live. No deferral period is applied after immunization with non-live vaccines. None of these regulations have a well-defined established safetynet. Hence, a precautionary measure ofdonating blood 14–28 days post vaccine deferral period is recommended at mostplaces [11]. Once the safety of COVID-19 vaccine on blood transmission is established, healthy asymptomatic individuals can donate blood more frequently with less deferral rates. This is important to prevent acute deficit due to surge in COVID-19 cases leading to drop in storage products.

Implications of reduced blood and its products will prove catastrophic, endangering a cohort of patients, if not dealt with timely. Maternal complications, major surgeries, hemorrhage, severe anemia, and a plethora of blood dyscrasias such as leukemia and sickle cell anemia among others, all necessitate the urgent requirement of blood and its components [12]. Availability of blood has been one of the main reasons for reducing the rates of maternal mortality [13]. It is therefore imperative that there are at least minimum adequate blood reserves to deal with life threatening situations. Selective blood banks and hospitals could be subjected to further strain as a result of outbreaks due to other diseases. Due to a surge in the COVID-19 cases and as a consequence of reduced blood supplies, several centers have also resorted to minimizing, postponing, or canceling electively scheduled operations, endoscopies, or other invasive procedures. In the future as hospitals eventually resume elective surgery, they will most likely encounter substantial number of surgical admissions, leading to long-term shortages which will probably persist even after the conclusion of the pandemic [14]. Besides an acute shortage of blood supplies, there also exists a dearth of testing agents and consumables as the laboratories are burdened with COVID-19 RT-PCR samples [15].

Several strategies could possibly be employed to prevent an acute shortage. Prioritizing policies and contingency planning would lay the framework for effective management of blood and its products. Blood donation, an essential service should be regarded as a permissible activity even during lockdown throughout the country. The over all deferral period after SARS-CoV-2 vaccination can possibly be reduced with strict hemovigilance systems to monitor potential causes of transfusion transmission [7].

Shortage of inventory supplies could be prevented by conducting regular updates of the available stock closely, and by monitoring activities that require increased use of available inventory such as elective surgeries [16]. During the pandemic, individuals seeking prior appointments for blood donation should be encouraged while walk-ins dissuaded [17]. In times of uncertainty, a few procedures and criteria with regards to donor safety could be considered for relaxation depending on the demand. For an instance, physical distribution of the certificate of appreciation could be replaced virtually. An attempt to increase the number of blood drives, can also help provide a short-term buffer in cases of immediate requirement in the early future [18].

It is also of utmost priority to strictly adhere to public health recommendations during the pandemic. Guidelines for COVID-19 signs and symptoms should be disseminated among the masses with proper screening, monitoring and epidemiological surveillance [19]. In cases of essential workers being tested positive for SARS-CoV-2, contingency planning for staff replacement (reassignment and training of other non-essential staff), as well as strengthening psychological support of affected as well as for new personnel will prove beneficial [20].

On a concluding note, the crisis of blood shortage amidst the ongoing pandemic needs a collaborative effort from the various stakeholders ranging from blood donors, community health workers, blood transfusion services, and administration in order to protect and save more lives.

## Ethical approval

NA.

## Please state any sources of funding for your research

NA.

## Author contribution

All the authors contributed equally for study concept or design, data collection, data analysis or interpretation and writing the paper. **Correspondin Author**: Utkarsha Uday.**Affiliation:** West Bengal University of Health Sciences, Kolkata, India.**Email**: utkarsha.tanuday@gmail.com.**Contact no**.: +91-7991156485.

## Please state any conflicts of interest

NA.

## Consent

NA.

## Registration of research studies

NA.

1. Name of the registry:

2. Unique Identifying number or registration ID:

3. Hyperlink to your specific registration (must be publicly accessible and will be checked):

## Guarantor

Utkarsha Uday.
